# Cognitive Outcomes in Pediatric Arteriovenous Malformation with Cerebral Hemorrhage: A Case Series

**DOI:** 10.3390/children13091207

**Published:** 2026-09-07

**Authors:** Tomoko Uchida, Daisuke Matsuzawa, Ryo Tanabe, Kasumi Nagasawa, Mitsuko Ishii, Michiyo Ehara, Tadashi Shiohama, Hiromichi Hamada

**Affiliations:** 1 Chiba Rehabilitation Center, Chiba 260-0005, Japan; ryo.tanabe@chiba-reha.jp (R.T.); kasumi-n@rk2.so-net.ne.jp (K.N.); mitsuko.ishii@chiba-reha.jp (M.I.); michiyo.ehara1831@ezweb.ne.jp (M.E.); 2 Department of Pediatrics, Graduate School of Medicine, Chiba University, Chiba 260-0856, Japan; shiohama-tadashi-vx@ihwg.jp (T.S.); hamada.hiromichi@chiba-u.jp (H.H.); 3 Research Center for Child Mental Development, Chiba University, Chiba 260-0856, Japan; dmatsuzawa@faculty.chiba-u.jp

**Keywords:** case report, arteriovenous malformation, pediatric, hemorrhage, cognitive function, rehabilitation

## Abstract

**Highlights:**

**What are the main findings?**

- Long-term cognitive impairment was frequent (60% with persistent dysfunction) in pediatric arteriovenous malformation (AVM) survivors, despite favorable motor recovery (90% achieving independent walking).
- Of the 8 patients whose Full-Scale IQ (FIQ) reached 80 or above within 6 months of onset, 6 went on to resume their premorbid educational trajectory, compared with 2 of the 12 whose early FIQ remained below 80.

**What are the implications of the main findings?**

- Neuropsychological assessment within 6 months of hemorrhage may serve as a practical prognostic anchor for educational and vocational planning, rather than awaiting prolonged spontaneous recovery.

**Abstract:**

**Background**: Arteriovenous malformation (AVM) is a leading cause of pediatric hemorrhagic stroke. While immediate management and short-term outcomes have been well-documented, research on long-term cognitive recovery after AVM hemorrhage remains limited, and outcomes are often difficult to predict. **Methods**: We report the long-term cognitive recovery outcomes of 20 pediatric cases (12 boys, 8 girls) who underwent rehabilitation and cognitive function assessments during the recovery phase at our center following acute treatment for intracranial hemorrhage due to AVM. The mean age at onset was 11.0 ± 3.1 years (range: 6.3–16.6 years). The primary outcome was resumption of the premorbid educational trajectory, and we evaluated its relationship with the initial Full-Scale IQ (FIQ) recorded after onset. **Results**: Motor recovery was favorable (90% achieved independent walking), but cognitive outcomes were generally poor, with 60% exhibiting persistent dysfunction. Eight patients (40%) resumed their premorbid educational trajectory. Of the 8 patients whose FIQ reached 80 or above within the first 6 months post-onset, 6 (75%) subsequently resumed that trajectory, compared with 2 of the 12 patients whose early FIQ remained below 80 (odds ratio 15.0, 95% CI 1.65–136.2, *p* = 0.019; sensitivity 75%, specificity 83%). A normal-range FIQ was necessary but not sufficient: all 8 patients who resumed their premorbid trajectory reached an FIQ ≥ 80, yet 4 of the 12 patients who reached an FIQ ≥ 80 did not resume it. **Conclusions**: Although FIQ continued to improve for years in many patients, late gains were rarely sufficient to reach the normal range when the early assessment was low. Cognitive status in the early recovery phase was therefore associated with long-term educational continuity, and assessment within the first 6 months post-onset may inform planning without awaiting prolonged spontaneous recovery.

## 1. Introduction

Arteriovenous malformation (AVM) is a leading cause of pediatric hemorrhagic stroke, accounting for up to 30–50% of cases [1], and is the most common cause of pediatric intracerebral hemorrhage in children aged 2 to 18 years [2]. AVMs are congenital anomalies derived from maldevelopment of the capillary network, in which a nidus of abnormal vessels forms a direct transition between arteries and veins without intervening capillaries, predisposing to rupture [3]. Most are sporadic, although a minority arise in association with hereditary hemorrhagic telangiectasia or other genetic conditions predisposing to vascular malformation [2]. While the immediate management and short-term outcomes have been well-documented, research on long-term cognitive recovery after AVM hemorrhage remains limited and is drawn largely from small, heterogeneous retrospective samples [4,5,6], and outcomes are often difficult to predict.

The developing brain possesses remarkable neuroplasticity, theoretically favoring better recovery in children compared to adults [7]. However, this recovery may be uneven across various domains, with motor functions often recovering more completely than cognitive abilities [5,7,8]. Executive functions are particularly vulnerable to disruption following brain injury in childhood, as demonstrated in recent neuropsychological profiles of AVM patients [4,9].

Previous studies of pediatric AVM have focused predominantly on mortality, obliteration rates, and motor outcome, and long-term cognitive trajectories have rarely been tracked beyond the first year. This case series examines long-term cognitive and motor recovery trajectories in 20 pediatric patients with AVM-related cerebral hemorrhage, all of whom had been functioning at mainstream academic levels prior to hemorrhage, and explores whether early cognitive status is associated with resumption of the premorbid educational trajectory.

## 2. Case Series Description

### 2.1. Study Setting and Patients

We retrospectively analyzed 20 pediatric cases (12 boys, 8 girls) with AVM-related cerebral hemorrhage followed at Chiba Rehabilitation Center between May 1988 and July 2016. All cases were confirmed by neuroimaging and underwent neuropsychological assessment during their recovery period. The mean age at onset was 11.0 ± 3.1 years (range: 6.3–16.6 years), and patients were followed up for a mean of 54.8 ± 42.4 months (range: 9–147 months). All patients had completed acute-phase treatment at other institutions before referral, and the accompanying clinical and imaging information varied in completeness; angioarchitectural and acute-care detail could not be ascertained consistently. Prognostic assessment in this setting therefore rests on what can be observed directly during the recovery phase, which is the perspective this series adopts.

### 2.2. Cognitive and Motor Assessment

Cognitive function was assessed using the Japanese edition of the Wechsler Intelligence Scale for Children, Third Edition (WISC-III), in 18 patients, from which Full-Scale IQ (FIQ), Verbal IQ (VIQ), and Performance IQ (PIQ) were derived. One patient who had reached 16 years old by the time of assessment was evaluated with the Wechsler Adult Intelligence Scale, Third Edition (WAIS-III), and one patient was assessed with the WISC, Fourth Edition (WISC-IV). For these two cases, the Full-Scale IQ was treated as equivalent to the WISC-III FIQ given the comparable normative scaling (mean 100, SD 15); for the WISC-IV case, the Verbal Comprehension and Perceptual Reasoning indices were used in place of Verbal and Performance IQ. Motor function was assessed by certified physical therapists; independent ambulation was defined as the ability to walk without physical assistance.

### 2.3. Outcome Definition

The primary outcome was resumption of the premorbid educational trajectory at final follow-up. No validated instrument exists for this construct in pediatric hemorrhagic stroke [6]; the definition used here was therefore developed by the authors and required all of the following: (i) return to a mainstream class at the grade level the patient would have been attending had the hemorrhage not occurred; (ii) no requirement for additional special educational support; and (iii) progression to post-compulsory education, vocational training, or employment consistent with the patient’s own stated preference.

Early cognitive status was defined as the initial FIQ documented after onset. This assessment was obtained within 6 months of onset in 15 of the 20 patients; in 4 patients the earliest available assessment fell outside this window (at 8, 9, 10, and 16 months), and in 1 patient no assessment was available before the 1–5-year follow-up window. The last patient was conservatively classified as not having met the early FIQ ≥ 80 criterion, and both of these departures were examined in sensitivity analyses.

### 2.4. Ethics

The study was conducted in accordance with the Declaration of Helsinki and was approved by the Ethics Committee of Chiba Rehabilitation Center (Medical Committee No. 8–11; date of approval, 18 June 2026).

### 2.5. Statistical Analysis

The association between early FIQ (≥80 versus <80) and resumption of the premorbid educational trajectory was tested with Fisher’s exact test; the odds ratio is reported with a 95% confidence interval derived by Woolf’s logit method. Sensitivity, specificity, and positive and negative predictive values were calculated with Wilson score confidence intervals, and the discriminative performance of early FIQ as a continuous variable was summarized by the area under the receiver operating characteristic curve. Two prespecified sensitivity analyses were performed: one excluding the patient without an early assessment, and one restricted to the 15 patients assessed within 6 months of onset. Given the sample size, no multivariable modelling was attempted; all analyses are descriptive and exploratory, and no adjustment was made for the acute clinical variables that were unavailable in this cohort. Two-sided *p* < 0.05 was considered significant.

## 3. Outcomes

### 3.1. Patient Characteristics

The hemorrhage locations included basal ganglia/thalamus (*n* = 5, 25%), frontal lobe (*n* = 4, 20%), temporal lobe (*n* = 3, 15%), parietal lobe (*n* = 3, 15%), cerebellum (*n* = 3, 15%), and occipital lobe (*n* = 1, 5%). One case (5%) had unspecified right hemisphere hemorrhage. Overall, 17 cases (85%) had supratentorial hemorrhage and 3 cases (15%) had infratentorial hemorrhage. Ten patients had left-hemispheric, 7 right-hemispheric, and 3 cerebellar or otherwise non-lateralized hemorrhage; the modest left-sided excess is within the range expected by chance in a series of this size. Among the left-hemispheric cases, 6 (60%) developed aphasia. While aphasia correlated with left hemisphere lesions, other cognitive dysfunctions showed no clear relationship with hemorrhage location. Patient characteristics are summarized in Table 1; per-patient data are provided in Table 2.

### 3.2. Long-Term Outcomes and Early Cognitive Status

At the latest follow-up (mean: 54.8 months), 18 patients (90%) achieved independent walking, and 2 (10%) were unable to walk independently. In contrast, cognitive outcomes were less favorable. Of the 20 patients who underwent Wechsler assessment, the mean Full-Scale IQ (FIQ) was 82.1 ± 15.5, with a mean Verbal IQ (VIQ) of 89.7 ± 14.5 and Performance IQ (PIQ) of 76.0 ± 17.0. Eight patients (40%) resumed their premorbid educational trajectory as defined in Section 2.3. This group demonstrated markedly higher IQ scores (FIQ 96.5 ± 7.0; VIQ 99.4 ± 5.7; PIQ 92.0 ± 9.9) than those who did not (*n* = 12; FIQ 72.5 ± 11.5; VIQ 83.2 ± 15.1; PIQ 65.4 ± 11.4). Of the 8 patients with an early FIQ ≥ 80, 6 (75%) subsequently resumed their premorbid educational trajectory; of the 12 patients with an early FIQ < 80, only 2 (17%) did so despite long-term rehabilitation. This association was statistically significant (Fisher’s exact test: OR = 15.0, 95% CI 1.65–136.2, *p* = 0.019). Treating an early FIQ ≥ 80 as a prognostic test yielded a sensitivity of 75.0% (95% CI 40.9–92.9), specificity 83.3% (55.2–95.3), positive predictive value 75.0% (40.9–92.9), and negative predictive value 83.3% (55.2–95.3); analyzed as a continuous variable, early FIQ discriminated the two outcome groups with an area under the receiver operating characteristic curve of 0.92 (*n* = 19 with an early assessment). The association was robust to the two departures noted in Section 2.3: excluding the patient without an early assessment gave an OR of 13.5 (95% CI 1.47–123.7, *p* = 0.024), and restricting the analysis to the 15 patients assessed within 6 months gave an OR of 18.0 (95% CI 1.27–255.8, *p* = 0.041).

A Full-Scale IQ in the normal range was necessary but not sufficient for this outcome. All 8 patients who resumed their premorbid educational trajectory reached a best FIQ ≥ 80 (Table 2); however, 4 of the 12 patients who reached a best FIQ ≥ 80 (cases 8, 9, 10, and 20) did not resume it. Psychometric recovery and educational continuity were therefore not interchangeable in this cohort.

**Figure 1:**
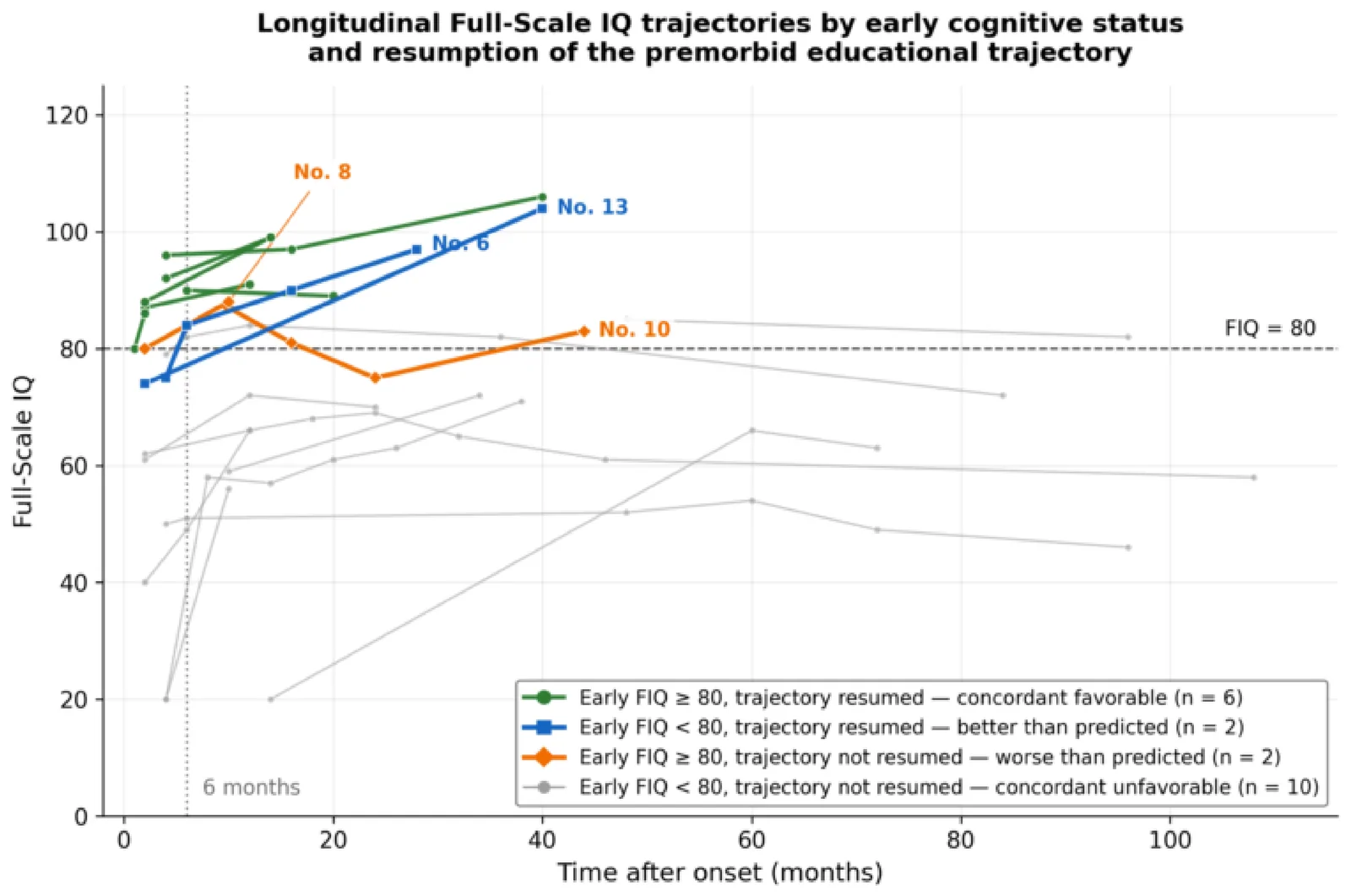
Longitudinal Full-Scale IQ (FIQ) trajectories of the 20 patients, grouped by early cognitive status and by resumption of the premorbid educational trajectory. Each line represents one patient. Green: early FIQ ≥ 80 and trajectory resumed (concordant favorable, *n* = 6). Blue: early FIQ < 80 yet trajectory resumed (better than predicted; cases 6 and 13). Orange: early FIQ ≥ 80 yet trajectory not resumed (worse than predicted; cases 8 and 10). Gray: early FIQ < 80 and trajectory not resumed (concordant unfavorable, *n* = 10). The horizontal dashed line marks FIQ = 80 and the vertical dotted line marks 6 months after onset. The four discordant cases are labeled with their case numbers.

Figure 1 illustrates the longitudinal trajectories of Full-Scale IQ scores for individual patients from the time of onset through follow-up.

## 4. Discussion

This case series demonstrates a notable discrepancy between motor and cognitive recovery outcomes in pediatric AVM patients. While 90% achieved independent ambulation at final follow-up, only a minority (40%) resumed their premorbid educational trajectory. This disparity aligns with recent research [7,8], highlighting the differential vulnerability of cognitive versus motor functions.

Our most clinically relevant observation was the relationship between early cognitive performance and long-term outcomes. Six of the eight patients whose IQ reached 80 or above within 6 months post-hemorrhage went on to resume their premorbid educational trajectory, compared with two of the twelve whose early IQ remained below 80. Although rapid recovery during the first year followed by a plateau has been documented in children with acquired brain injury [10,11], Blom et al. reported that only one-quarter of childhood hemorrhagic stroke survivors were free of both physical and cognitive deficits at long-term follow-up, with a decline in cognitive efficiency observed even in those whose IQ had recovered to the average range [4]. This accords with the recent consensus statement of the International Pediatric Stroke Organization, in which intellectual function emerged as the most robust predictor of the long-term need for special educational support after pediatric hemorrhagic stroke [6]. However, to our knowledge, no study has longitudinally tracked IQ trajectories over an extended follow-up period specifically in pediatric patients with AVM-related hemorrhage.

Figure 1 shows that FIQ continued to rise in many patients well beyond the first year, and this deserves comment. Between the initial and the best subsequent assessment, FIQ improved by a mean of 9.4 points (range −1 to +30). Crucially, the magnitude of improvement was similar in both outcome groups (+10.8 versus +8.5 points), but the endpoints were not: patients who resumed their premorbid trajectory reached a mean best FIQ of 96.5, whereas those who did not reached only 72.5. Late gains were therefore real and consistent with the post-acute improvement in global outcome reported by Dinc et al. [7], but they were largely parallel rather than compensatory, and rarely sufficient to reach the normal range when the early assessment was low. The practical implication is not that recovery ceases at 6 months, but that waiting for it carries a cost: a child whose FIQ has not approached the normal range by 6 months is unlikely to return to their previous educational course without sustained additional support, and planning for that support need not be deferred.

The outcome used here is not a validated instrument—none exists specifically for pediatric hemorrhagic stroke [6]—and the proportion classified as favorable depends on how the outcome is framed. Twelve patients (60%) eventually reached an FIQ ≥ 80, yet only 8 (40%) resumed their premorbid educational trajectory; the four patients in whom these classifications diverged illustrate that a normal-range IQ does not guarantee educational continuity. We emphasize that the latter is a descriptive marker of whether the hemorrhage displaced a child from the course they were already on, and not a judgment of ability: children who require special educational support or who follow an alternative pathway may achieve entirely satisfactory vocational and personal outcomes. Educational attainment is in addition shaped by socioeconomic circumstances, family support, regional differences in the availability of special education, and changes in Japanese educational policy over the study period, none of which could be accounted for here.

Our findings have several important clinical implications. First, comprehensive neuropsychological evaluation should be prioritized in the early recovery phase, ideally within 6 months post-hemorrhage, as this assessment appears to carry substantial prognostic value. Second, patients with early FIQ scores below 80 may represent a high-risk subgroup requiring more intensive, targeted cognitive intervention and educational support, rather than a “wait-and-see” approach. While the two patients in that group who nonetheless resumed their trajectory suggest the possibility of intellectual recovery, the remaining 10 continued to face significant challenges in daily life and to require specialized education or significant accommodations. Dinc et al. reported that although 88.9% of young patients with ruptured AVMs returned to work or school during long-term follow-up, cognitive problems persisted [7]. Thus, long-term follow-up is essential even for patients with good physical recovery, as cognitive deficits often persist and affect academic performance and quality of life.

A further implication concerns the setting in which prognostic judgments are made. Children reaching rehabilitation services have usually been treated elsewhere, and the detail available about the lesion is variable, as it was here; prognostic assessment must then rest on what can be observed directly. Irrespective of AVM morphology, initial severity, complications, and treatment modality—none of which we could characterize—cognitive status at 6 months was associated with whether a child subsequently resumed their premorbid educational trajectory. If confirmed in larger cohorts, this would allow support to be planned at around 6 months rather than after the one to two years that a wait-and-see approach entails. Because the acute variables were unavailable, we cannot say whether early FIQ carries prognostic information beyond them or merely summarizes their combined effect; for the clinician who must plan support without that information, the distinction may matter less than the fact that a 6-month assessment is informative at all.

This study has several limitations. It was retrospective and small, accrued at a single rehabilitation center, and is subject to referral bias in both directions: children with mild deficits may never have been referred, while the most severely affected may not have survived or been testable. Neuropsychological data were abstracted from routine clinical records rather than collected to a common protocol, the primary outcome is not a validated instrument, and follow-up varied widely (9–147 months), so that patients assessed over a shorter period may have improved further. Surgical and endovascular practice, imaging, assessment instruments, and educational provision all changed over the accrual period, and acute clinical variables were not consistently available (Section 2.1); our findings therefore describe an association between early cognitive status and later educational continuity rather than establishing early FIQ as an independent predictor. Prospective multicenter studies with a common protocol and fixed follow-up intervals are needed; given the rarity of pediatric AVM, however, series such as this remain a necessary starting point.

## 5. Conclusions

Pediatric patients with AVM-related hemorrhage demonstrated good motor recovery but persistent cognitive impairment. Cognitive status in the early recovery phase was associated with whether children later resumed their premorbid educational trajectory, and although IQ continued to improve for years, late gains were rarely sufficient to reach the normal range when the early assessment was low. Within the limits of a small retrospective series lacking acute clinical variables, neuropsychological assessment in the first 6 months may therefore help identify children who will need sustained educational support, rather than deferring that judgment until spontaneous recovery has run its course.

## Figures and Tables

**Table 1:** Demographic, clinical, and cognitive characteristics of the 20 pediatric patients with arteriovenous malformation-related cerebral hemorrhage.

Variable	Value (*n* = 20)
Sex, male/female, *n*	12/8
Age at onset, years, mean ± SD	11.0 ± 3.1
Age at onset, years, range	6.3–16.6
Handedness, right/left, *n*	19/1
Follow-up, months, mean ± SD	54.8 ± 42.4
Follow-up, months, range	9–147
**Hemorrhage location, *n* (%)**	
Basal ganglia/thalamus	5 (25%)
Frontal lobe	4 (20%)
Temporal lobe	3 (15%)
Parietal lobe	3 (15%)
Cerebellum	3 (15%)
Occipital/other	2 (10%)
**Hemorrhage compartment**	
Supratentorial	17 (85%)
Infratentorial	3 (15%)
**Hemorrhage side**	
Left	10 (50%)
Right	7 (35%)
Cerebellar/non-lateralized	3 (15%)
**Motor function at final follow-up**	
Independent walking	18 (90%)
Non-independent	2 (10%)
Aphasia (in left-hemispheric cases)	6/10 (60%)
**Best WISC scores 1–5 years after onset (*n* = 20), mean ± SD**	
FIQ	82.1 ± 15.5
VIQ	89.7 ± 14.5
PIQ	76.0 ± 17.0
**Early recovery group (FIQ ≥ 80 at ≤6 months)**	
*n* (% of cohort)	8 (40%)
FIQ	93.2 ± 7.2
VIQ	97.6 ± 5.9
PIQ	87.9 ± 10.0
**Non-early recovery group (FIQ < 80 at ≤6 months)**	
*n* (% of cohort)	12 (60%)
FIQ	74.7 ± 15.2
VIQ	84.4 ± 16.3
PIQ	68.2 ± 16.4
**Premorbid educational trajectory resumed at final follow-up**	
Achieved	8 (40%)
Not achieved	12 (60%)

FIQ = Full-Scale IQ; VIQ = Verbal IQ; PIQ = Performance IQ; WISC = Wechsler Intelligence Scale for Children.

**Table 2:** Demographic, clinical, and cognitive characteristics of the 20 individual pediatric patients with arteriovenous malformation-related cerebral hemorrhage, color-coded by prognostic category.

Case No.	Sex	Age at Onset	Handedness at Onset	Follow-Up Period (Months)	Main Hemorrhage Site	Best WISC-III Scores 1–5 Years After Onset: FIQ, VIQ, PIQ (Months After Onset)
1	M	7y11mo	right	113	Left basal nuclei	54, 58, 60 (65)
2	F	9y10mo	right	82	Left temporal lobe	71, 70, 78 (38)
3	F	9y9mo	right	109	Right cerebellum	66, 67, 61 (57)
4	M	7y6mo	right	147	Left parietal lobe	69, 95, 47 (24)
5	F	11y2mo	right	92	Right hemisphere	72, 84, 65 (11)
6	F	15y5mo	right	28	Cerebellum	97, 102, 91 (28)
7	F	12y1mo	right	25	Right frontal lobe	56, 65, 55 (7)
8	F	14y10mo	right	10	Right frontal lobe	88, 92, 86 (10)
9	F	7y6mo	right	97	Right temporal lobe	84, 103, 66 (12)
10	M	8y4mo	right	43	Left cerebellum	87, 99, 76 (10)
11	F	8y10mo	right	25	Left frontal lobe	91, 97, 86 (11)
12	M	10y9mo	right	21	Left putamen	99, 100, 99 (13)
13	M	16y7mo	right	72	Left parietal lobe	104, 103, 104 (39): WAIS-III
14	M	7y7mo	left	40	Left frontal lobe	106, 108, 103 (40)
15	M	14y10mo	right	9	Right thalamus	86, 95, 85 (3): WISC-IV
16	M	10y11mo	right	35	Right basal nuclei	72, 91, 55 (34)
17	M	14y6mo	right	20	Left parietal lobe	90, 89, 93 (6)
18	M	12y5mo	right	14	Left basal nuclei	99, 101, 75 (14)
19	M	13y5mo	right	13	Right temporal lobe	66, 79, 60 (13)
20	M	6y4mo	right	100	Left occipital lobe	85, 96, 76 (52)
**Prognostic category (row color):**						
	Early FIQ ≥ 80 and premorbid trajectory resumed—concordant favorable (*n* = 6).					
	Trajectory resumed despite early FIQ < 80—better than predicted (No. 6 and 13).					
	Early FIQ ≥ 80 but trajectory not resumed—worse than predicted (No. 8 and 10).					
	Early FIQ < 80 and trajectory not resumed—concordant unfavorable (*n* = 10).					

FIQ ≥ 80 = Full-Scale IQ ≥ 80 documented within the first 6 months post-onset. Resumption of the premorbid educational trajectory = return to a mainstream class at the grade level the patient would have been attending had the hemorrhage not occurred, no requirement for additional special educational support, and progression to a post-compulsory pathway consistent with the patient’s own preference, at final follow-up. FIQ = Full-Scale IQ; VIQ = Verbal IQ; PIQ = Performance IQ; WISC-III = Wechsler Intelligence Scale for Children, Third Edition; WAIS-III = Wechsler Adult Intelligence Scale, Third Edition; WISC-IV = Wechsler Intelligence Scale for Children, Fourth Edition. Cognitive scores are the best Full-Scale, Verbal, and Performance IQ values obtained 1–5 years after onset, with the timing of assessment (months after onset) in parentheses. For the WISC-IV case (No. 15), the reported values are FSIQ and the VCI/VIQ- and PRI/PIQ-equivalent indices.

## Data Availability

The data presented in this study are available on reasonable request from the corresponding author. The data are not publicly available due to privacy restrictions involving pediatric clinical records.
